# Avian olfactory receptor gene repertoires: evidence for a well-developed sense of smell in birds?

**DOI:** 10.1098/rspb.2008.0607

**Published:** 2008-07-15

**Authors:** Silke S Steiger, Andrew E Fidler, Mihai Valcu, Bart Kempenaers

**Affiliations:** 1 Department of Behavioural Ecology and Evolutionary Genetics, Max-Planck Institute for OrnithologyPO Box 1564, 82319 Starnberg, Germany; 2 Cawthron InstitutePrivate Bag 2, Nelson 7042, New Zealand

**Keywords:** olfaction, bird, olfactory bulb, pseudogene, nocturnality, ecological adaptation

## Abstract

Among vertebrates, the sense of smell is mediated by olfactory receptors (ORs) expressed in sensory neurons within the olfactory epithelium. Comparative genomic studies suggest that the olfactory acuity of mammalian species correlates positively with both the total number and the proportion of functional OR genes encoded in their genomes. In contrast to mammals, avian olfaction is poorly understood, with birds widely regarded as relying primarily on visual and auditory inputs. Here, we show that in nine bird species from seven orders (blue tit, *Cyanistes caeruleus*; black coucal, *Centropus grillii*; brown kiwi, *Apteryx australis*; canary, *Serinus canaria*; galah, *Eolophus roseicapillus*; red jungle fowl, *Gallus gallus*; kakapo, *Strigops habroptilus*; mallard, *Anas platyrhynchos*; snow petrel, *Pagodroma nivea*), the majority of amplified OR sequences are predicted to be from potentially functional genes. This finding is somewhat surprising as one previous report suggested that the majority of OR genes in an avian (red jungle fowl) genomic sequence are non-functional pseudogenes. We also show that it is not the estimated proportion of potentially functional OR genes, but rather the estimated total number of OR genes that correlates positively with relative olfactory bulb size, an anatomical correlate of olfactory capability. We further demonstrate that all the nine bird genomes examined encode OR genes belonging to a large gene clade, termed γ-c, the expansion of which appears to be a shared characteristic of class Aves. In summary, our findings suggest that olfaction in birds may be a more important sense than generally believed.

## 1. Introduction

Olfactory receptors (ORs) expressed in sensory neurons within the olfactory epithelium constitute the molecular basis of the sense of smell among vertebrates (Buck & Axel 1991; Gaillard *et al*. 2003). OR genes are small (approx. 1000 bp), intronless (Young & Trask 2002; Mombaerts 2004) and are thought to evolve rapidly, following a ‘birth-and-death’ model (Nei *et al*. 1997). Both the size of the OR gene family and the proportion of OR genes that are non-functional (i.e. pseudogenes) vary widely between vertebrate genomes (size range: 100–2130 in pufferfish, *Fugu rubripes*, and cow, *Bos taurus*, respectively; predicted functional proportion range: 40–80% in human and mouse, respectively; Mombaerts 2004; Niimura & Nei 2006, 2007). Comparative genomic studies suggest that the olfactory acuity of mammalian species correlates positively with both the total number and the proportion of functional OR genes encoded in their genomes (Rouquier *et al*. 2000; Gilad *et al*. 2004; Niimura & Nei 2006, 2007). The total number of OR genes in a genome may reflect how many different scents can be detected and distinguished (Niimura & Nei 2006). The proportion of functional OR genes provides insights into the selective pressures that have acted on the OR genes (Rouquier *et al*. 2000; Niimura & Nei 2006). For example, if olfaction has become less important during the evolutionary history of a species, an associated relaxation of conservative selection pressure may have led to an increase in the number of pseudogenes (i.e. no selection against loss-of-function mutations). Indeed, it has been suggested that a decline in the proportion of functional OR genes in the human genome is associated with a less keen sense of smell when compared with other primates (Rouquier *et al*. 2000; Gilad *et al*. 2004). OR genes have been studied extensively in fishes and mammals (Niimura & Nei 2006). By contrast, far less is known about avian OR genes. This may reflect the general belief that birds lack a well-developed sense of smell, although behavioural studies have shown that some bird species use their sense of smell to navigate (Papi 1991), forage (Wenzel 1968; Nevitt *et al*. 2008) or distinguish individuals (Bonadonna & Nevitt 2004; for reviews, see Roper 1999; Hagelin 2006; Hagelin & Jones 2007).

To date, avian OR gene sequence data have been limited to the domestic chicken (*Gallus gallus domesticus*) and its wild progenitor, the red jungle fowl (*Gallus gallus*; Leibovici *et al*. 1996; Nef *et al*. 1996; International Chicken Genome Sequencing Consortium 2004; Niimura & Nei 2005; Lagerström *et al*. 2006; but see Eriksson *et al*. 2008). An analysis of a draft (Build v. 1.1) *G. gallus* genomic sequence reported that (i) the OR gene repertoire consists of approximately 550 members, (ii) the predicted proportion of potentially functional OR genes was approximately 15%, and (iii) the majority of the *G. gallus* OR genes clustered within a single large clade, denoted group-γ-c (Niimura & Nei 2005). The group-γ-c clade appears to have expanded in size after the separation of the avian and mammalian lineages (Niimura & Nei 2005) and represents an expansion of OR genes similar to the human OR5U1 and OR5BF1 genes (International Chicken Genome Sequencing Consortium 2004; Lagerström *et al*. 2006). Note that, because the *G. gallus* genomic sequence analysed (Build v. 1.1) was of draft status, the estimated number and proportion of potentially functional OR genes should be considered as underestimates (Niimura & Nei 2005). Indeed, other studies estimated the potentially functional OR gene repertoire of the Build v. 1.1 draft *G. gallus* genomic sequence to be either 229 (Lagerström *et al*. 2006) or 283 (International Chicken Genome Sequencing Consortium 2004). The surprisingly large difference in the estimated number of potentially functional OR genes identified in those studies may be attributed to the different bioinformatics search strategies used.

In this study, we estimated the proportion of potentially functional OR genes encoded within the *G. gallus* genome and within the following eight other, taxonomically diverse, bird genomes: the blue tit (*Cyanistes caeruleus*); the black coucal (*Centropus grillii*); the brown kiwi (*Apteryx australis*); the canary (*Serinus canaria*); the galah (*Eolophus roseicapillus*); the kakapo (*Strigops habroptilus*); the mallard (*Anas platyrhynchos*); and the snow petrel (*Pagodroma nivea*). We further investigated whether either the proportion of potentially functional OR genes or the estimated total number of OR genes correlates with the olfactory bulb ratio (OBR), a possible anatomical correlate of olfactory capability (Edinger 1908). OBRs vary widely among avian species (Bang & Cobb 1968) and the nine species we investigated cover the entire range. Additionally, we estimated the total number of OR genes, both potentially functional and non-functional, in the nine species using a sample-coverage approach (Chao & Lee 1992). Finally, we derived phylogenetic trees from predicted OR protein sequences to test whether the recently expanded group-γ-c OR genes are specific to the red jungle fowl or are a shared characteristic of bird genomes.

## 2. Material and methods

### (a) Amplification and sequencing of OR genes

Blood samples were suspended in Queen's lysis buffer and stored at ambient temperature. Genomic DNA was isolated using a commercial kit (DNeasy tissue kit; Qiagen, Hilden, Germany) and approximately 100 ng was used as a template in subsequent amplification reactions. In total, 10 primers were designed to anneal to evolutionarily conserved coding sequences corresponding to the transmembrane (TM) domain 3 (forward primers) and TM7 (reverse primers) regions of the OR proteins. PCR primer pairs falling into two categories targeting either (i) the non-γ-c OR clade sequences or (ii) the γ-c OR clade sequences were used. To amplify non-γ-c OR sequences, three previously reported forward primers corresponding to the conserved TM3 amino acid sequences of (A)MAYDRY (5′-ATG GCI TAY GAY MGI TA-3′ and 5′-GCI ATG GCI TAY GAY MGI TA-3′; Nef *et al*. 1996; Freitag *et al*. 1999) and MAYDRY(V/L)AIC (5′-ATG GCI TAY GAY MGI TAY STI GCI ATY TG-3′; Leibovici *et al*. 1996) were paired with three reverse primers corresponding to the conserved TM7 amino acid sequences PMLNPLIY (5′-TA DAT IAG IGG RTT IAG CAT IGG-3′), NPFIYS(F/L) (5′-AR ISW RTA DAT RAA IGG RTT-3′; Freitag *et al*. 1999) and PM(L/F)NP (5′-GG RTT IAR CAT IGG-3′; Nef *et al*. 1996). Amplifications were conducted using each forward primer in combination with each reverse primer, thereby generating nine different PCR products. For the amplification of γ-c OR clade sequences, three forward primers corresponding to sequences found to be conserved among the reported red jungle fowl γ-c OR TM3 amino acid sequences ICKPLHY (5′-ATC TGY AAR CCI YTI CAY TA-3′) and VAICKPLHY (5′-ATC TGY AAR CCI YTI CAY TA-3′ and 5′-RTT GCI ATY TGY AAR CCY CTR CAC TA-3′) were used in combination with the reverse primer designed to the conserved TM7 amino acid OR sequence NPFIYS(F/L) (5′-AR ISW RTA DAT RAA IGG RTT-3′; Freitag *et al*. 1999).

All primer pairs were predicted to generate products of approximately 0.5 kb, which represents approximately half of the expected full OR-coding sequence. The PCR was carried out in a final volume of 50.0 μl, containing 2.0 mM Mg^2+^, dNTPs (0.1 mM), primers (0.8 μM), 1 U *Taq* DNA polymerase (MBI Fermentas, St Leon-Rot, Germany) and genomic DNA (100 ng) template with thermocycling parameters: 94°C/2 min; 94°C/30 s, 37°C/30 s, ramping from 37 to 72°C at 0.2°C s^−1^, 72°C/60 s, 5 cycles; 94°C/30 s, 45°C/30 s, 72°C/60 s, 30 cycles; 72°C/7 min; and 4°C/hold.

Amplification products were separated through 2% agarose gels (Nusieve GTG agarose, BioWhittaker Molecular Applications, Rockland, ME, USA), bands were excised and purified (QIAquick Gel Extraction kit, Qiagen) before ligation into a T-tailed cloning vector (pGemT-easy, Promega, Madison, WI, USA). Note that PCR products were not pooled for ligation and transformation. Plasmids having inserts were purified from transformed DH5α colonies by alkaline lysis (High Pure Plasmid Isolation kit; Roche Diagnostics, Mannheim, Germany) and sequenced by external contractors (MWG Biotech, Ebersberg, Germany; AGOWA, Berlin, Germany).

### (b) Sequence analysis and phylogenetic tree construction

We obtained on average 150±11 s.e.m. (range 98–206) sequences per species. Electropherograms were visually inspected, edited and low-quality sequences discarded. PCR primer sequences were deleted and sequences sharing more than or equal to 98.5% identity, determined using the ‘SEQUENCE IDENTITY MATRIX’ function of BioEdit (Hall 1999; http://www.mbio.ncsu.edu/bioedit/bioedit.html), were considered to be amplified from a single OR gene (Fuchs *et al*. 2001). This procedure was used to accommodate errors introduced by the amplification itself. It may contribute to an underestimation of the total OR gene number due to the clustering of highly similar, but distinct, paralogues. To confirm that the sequences were partial OR-coding sequences, each sequence was used as a query in a BLAST search in the NCBI's non-redundant database. The sequences that did not return an established vertebrate OR sequence as a ‘best hit’ were removed from further analyses. The sequences were shifted into the correct reading frame using a custom-written PERL script. Owing to the use of different primer pairs, OR fragments varied in length. Thus, we restricted deduced receptor protein sequences to appropriate length (Freitag *et al*. 1998). Amplified avian partial OR-coding sequences were classified as being either non-γ-c or γ-c on the basis of sequence homologies between their corresponding predicted proteins and 78 potentially functional red jungle fowl (*G. gallus*) OR sequences of established classification (Niimura & Nei 2005). Note that the OR genes amplified with the primers annealing to the conserved regions ICKPLHY/NPFIYS(F/L) or VAICKPLHY/NPFIYS(F/L) that did not belong to the γ-c clade were removed from the analysis. A summary of all the amplified partial OR-coding sequences and the corresponding primer combinations used is shown in table S1 of the electronic supplementary material. We assigned a sequence as a potentially functional gene if an uninterrupted coding region was found (i.e. sequence without stop codon) while, if an interrupted coding region was found (i.e. sequence with stop codon), the sequence was assigned as a pseudogene (Gilad *et al*. 2004). In nine cases, copies of what appeared to be the same sequence were both potentially functional and pseudogenes and these were excluded from further analysis. Note that this method may overestimate the proportions of potentially functional OR genes, because frame-shift mutations outside the amplified coding region or mutations in promoter regions will not be detected. To determine how many potentially ‘functional’ OR-coding sequences from the experiments are indeed pseudogenes (owing to mutations outside the amplified region), we conducted a search for OR genes in the second draft of the *G. gallus* genome (Build v. 2.1, May 2006 release). The *G. gallus* OR sequences identified with the PCR-based method were then compared against the set of OR genes identified by the search using a BLAST approach. In addition, we compared the *G. gallus* sequences based on the degenerate PCR approach with Niimura & Nei's (2005) dataset, which was based on the first draft of the *G. gallus* genome.

A generalized linear mixed model (GLMM) was used to compare the proportion of potentially functional OR genes between the γ-c and the non-γ-c clades (Venables & Ripley 2002). The number of potentially functional OR genes amplified was used as the dependent variable, the total number of amplified OR genes as the binomial denominator, the species as a random factor and the clade as a predictor variable.

Clustal X v. 1.81 (Thompson *et al*. 1997) was used with default parameters to construct multiple amino acid sequence alignments. The neighbour-joining (NJ) method was used to generate phylogenetic trees from Poisson correction distances using the Mega software (http://www.megasoftware.net/). The reliability of the phylogenetic tree was evaluated with 1000 bootstrap repeats.

### (c) Estimation of OR repertoire size

A non-parametric estimation technique applying the concept of ‘sample coverage’ (Chao & Lee 1992) was used to estimate the total number of OR genes in each of the nine avian genomes investigated. In a first step, the number of times identical PCR products were re-sequenced was used to estimate sample coverage (C) and its coefficient of variation (CV). In a second step, we chose the appropriate coverage estimator given the information provided by C and CV. This method does not assume an equal probability for each gene to be cloned and thus accounts for a primer bias. The black coucal was excluded from further analysis due to a large CV. Abundance coverage estimators, their standard errors, confidence intervals and related statistics for all species were calculated using the software Spade (http://chao.stat.nthu.edu.tw/) and can be found in table S2 of the electronic supplementary material. Note that the estimated total number of OR genes might be an underestimate of the true value (Bunge & Fitzpatrick 1993).

### (d) Phylogenetically independent contrasts

To control for phylogenetic non-independence, we calculated phylogenetically independent contrasts (Felsenstein 1985) using the PDAP:PDTree module of Mesquite (Midford *et al*. 2005; Maddison & Maddison 2006). The topology of the tree and branch lengths was obtained by using genetic distances derived from DNA–DNA hybridization studies (Sibley & Ahlquist 1991; see figure S1 of the electronic supplementary material). Since we could not estimate the number of OR genes from the black coucal (see §2*c*), we obtained seven contrasts from the eight species.

## 3. Results

### (a) Proportion of potentially functional OR genes

We amplified 46 distinct partial OR-coding sequences from red jungle fowl (*G. gallus*, order Galliformes) genomic DNA (table 1; table S1, electronic supplementary material). The large majority (95.7%) of the partial OR-coding sequences was predicted to be amplified from potentially functional OR genes. To determine whether this high potentially functional/non-functional ratio is a general characteristic of bird genomes, we amplified between 26 and 68 (mean±s.e.m. 53.5±4.2) partial OR-coding sequences from a further eight species representing six additional avian orders (table 1; table S1, electronic supplementary material). The estimated proportion of potentially functional OR genes was consistently high in all taxa (mean±s.e.m. 83.7%±2.3%) despite the wide phylogenetic distribution and diverse ecological niches of the taxa examined (table 1). The estimated proportion of potentially functional OR genes was not statistically significantly different between the large γ-c OR clade (mean±s.e.m. 80.8%±3.9%) and the non-γ-c OR clade (mean±s.e.m. 85.7%±2.7%; GLMM, *t*_1,8_=0.34, *p*=0.74).

### (b) Comparison of data based on degenerate PCR and genome search

Of the 46 *G. gallus* sequences that we amplified using the degenerate PCR method, 18 were identical (more than or equal to 98.5% nucleotide identity) to the OR genes identified from the *G. gallus* genome search (Build v. 2.1, May 2006 release). The other 28 sequences were on average 94.9±0.5% identical to the OR genes identified from the *G. gallus* genome search. Because the large majority of the other sequences (27 out of 28) mapped to ‘chrUn_random’ regions in the *G. gallus* genome and because the Build v. 2.1 genome draft still contains many sequence gaps, we assume that we amplified many OR-coding sequences that are not yet in the Build v. 2.1 genomic sequence.

A direct comparison of the results from the degenerate PCR and from the genome search showed that two coding sequences which were identified as potentially functional with the PCR-based method turned out to be pseudogenes due to mutations outside the amplified region. Thus, we overestimated the proportion of potentially functional OR genes in the *G. gallus* genome by 11%. The comparison of the *G. gallus* sequences with Niimura & Nei's dataset yielded similar results (details not shown).

### (c) Numbers of avian OR genes and relationship with relative olfactory bulb size

The estimated total number of OR genes, both potentially functional and non-functional, varied widely among the nine species investigated (range 107–667; table 1; table S2, electronic supplementary material). Our estimate for the *G. gallus*, 638 OR genes, is close to the previously reported estimate of 550 derived by Niimura & Nei (2005) from a draft *G. gallus* genome sequence. This suggests that our methodology provides a reasonably reliable estimate of OR gene repertoire sizes in those species for which full genomic sequences are not yet available. The lower values (107–218; canary, blue tit and galah) are within the range reported as typical of fishes (Ngai *et al*. 1993), while the higher values (600–667; red jungle fowl, brown kiwi and kakapo) rather resemble those of mammalian genomes (Glusman *et al*. 2001).

The estimated total number of OR genes, but not the proportion of potentially functional OR genes, correlated positively with relative olfactory bulb size as measured by the OBR, the ratio of the greatest diameter of the olfactory bulb to the greatest diameter of the cerebral hemisphere in per cent (Bang & Cobb 1968; number: *r*=0.63, *n*=8, *p*<0.05 (one-tailed), figure S2*a*, electronic supplementary material; proportion: *r*=0.20, *n*=9, *p*=0.6, figure S2*b*, electronic supplementary material).

### (d) Phylogenetic trees derived from predicted OR protein sequences

An expanded γ-c OR clade is present in all the nine avian genomes examined (figure 1*a*). This clade was supported with a high bootstrap value (91%). Within this clade, there is a strong tendency for sequences from the same species, or species from the same order, to cluster together (figure 1*a*). By contrast, among the non-γ-c OR sequences, the overall pattern is one of intermingling of sequences from differing taxa, presumably, reflecting that these gene lineages diverged before the diversification of these avian orders. An NJ tree based on an alignment of the 405 predicted potentially functional avian OR protein sequences identified in this study (table 1) and the corresponding regions of potentially functional OR proteins identified from the red jungle fowl, zebrafish and human genome sequences (Niimura & Nei 2005) confirmed that the avian non-γ-c OR sequences intermingle with the other vertebrate OR protein sequences, whereas the avian γ-c OR clade sequences do not (figure 1*b*).

## 4. Discussion

Our results strongly suggest that the proportion of potentially functional OR genes in avian genomes is considerably higher than the value of 15% estimated from an analysis of the Build v. 1.1 draft red jungle fowl (*G. gallus*) genomic sequence by Niimura & Nei (2005). Our results are consistent with those of the International Chicken Genome Sequencing Consortium (2004), who estimated that 93% (202 out of 218) of OR genes in the γ-c OR clade are potentially functional (also based on the analysis of the Build v. 1.1 draft red jungle fowl genome sequence). In addition, our results are consistent with an unpublished analysis of a more recent draft of the *G. gallus* genome (Build v. 2.1, May 2006 release; V. Kuryshev, S. S. Steiger, M. Stensmyr, B. Kempenaers & J. Mueller 2008, unpublished data). In this analysis, the proportion of potentially functional OR genes was estimated to be at least 53%. Note that even in the Build v. 2.1 draft, many putative partial OR genes still contain sequence gaps, so it is likely that many will be classified as functional in subsequent drafts. Assuming that all such incomplete OR genes are functional, the proportion of potentially functional OR genes in the *G. gallus* genome could be as high as 85% (V. Kuryshev, S. S. Steiger, M. Stensmyr, B. Kempenaers & J. Mueller 2008, unpublished data).

The estimated total number of OR genes differed widely between the bird genomes studied (range 107–667), indicating that different ecological niches may have shaped the OR gene repertoires in birds, as has been suggested for mammals (Niimura & Nei 2007). The observed differences in OR gene repertoire sizes are striking, but perhaps not too surprising for the following two reasons. First, birds also show a wide interspecific variation in the relative olfactory bulb size, as quantified by the OBR. For example, the OBR of the snow petrel (*P. nivea*) is 12 times larger than that of the black-capped chickadee (*Poecile atricapillus*; Bang & Cobb 1968). Hence, a similar interspecific variation in OR gene repertoire size could be expected. Second, in mammals, OR gene repertoire sizes range from 606 OR genes in the macaque to 2129 OR genes in the cow (Niimura & Nei 2007). Thus, OR gene repertoire sizes also greatly vary among mammalian species.

We estimated both the total number and the proportion of potentially functional OR genes in the nine different avian genomes using PCR primers annealing to evolutionarily conserved regions. Because it is unlikely that full genomic data for more avian species will become available in the near future (with the exception of the Australian passerine zebra finch, *Taeniopygia guttata*), PCR using degenerate primers is currently the only available method to study the avian OR gene repertoires in an ecological context. This method has already been used to estimate the fraction of potentially functional OR genes in relatively poorly characterized genomes of primates (Rouquier *et al*. 2000; Gilad *et al*. 2004), carnivores (Quignon *et al*. 2003), rodents (Rouquier *et al*. 2000) and marine mammals (Kishida *et al*. 2007).

Notwithstanding its wide application, it is well recognized that this PCR-based approach has limitations and may overestimate the proportion of potentially functional OR genes, because (i) primer annealing sites may be more conserved in functional than in pseudogenes, and (ii) mutations that occur in regions not amplified by the primers will not be detected (Gilad *et al*. 2004). By comparing our PCR-based data with genome sequence information, we showed that the PCR-based approach overestimated the proportion of potentially functional OR-coding sequences in the *G. gallus* genome by approximately 11%. It is reasonable to assume that the extent of overestimation is similar for the other bird genomes.

Another disadvantage of the PCR-based method is that due to unpredictable primer bias, some OR genes may amplify preferentially. Thus, the ratios of partial OR-coding sequences among the amplification products may not represent a random sample of the OR repertoires in the genomes used as templates. However, if the primers were biased, we expect the bias to occur in all species and the between-species comparison should thus remain valid. Furthermore, it seems unlikely that a primer bias would generate a positive correlation between the estimated number of OR genes and the OBR. Finally, it has already been shown that PCR-based and whole-genome estimates yielded similar results. For example, Gilad *et al*. (2004) and Malnic *et al*. (2004) estimated the proportion of functional OR genes in humans to be approximately 48 and 53% in a PCR-based and genome-wide approach, respectively. Taken together, we argue that the PCR-based method is a useful approach to estimate the OR gene repertoires in birds.

Our results further suggest that estimating OR gene numbers in a wider range of avian genomes may provide insights into the selective pressures that have driven the evolution of avian olfaction. Ecological niche-associated adaptations such as daily activity pattern (e.g. nocturnal versus diurnal), habitat (e.g. terrestrial versus aquatic) or diet (e.g. generalist versus specialist) may well have shaped, and perhaps been driven by, the OR gene repertoires. For example, our finding that two night-active species, the kiwi and the kakapo, have comparatively large OR gene repertoires is consistent with the hypothesis that nocturnal species have evolved enhanced olfactory ability to deal with reduced effectiveness of vision under low-light conditions (Healy & Guilford 1990). The snow petrel seems to be an outlier in the sense that it has one of the largest OBRs measured in birds, but a relatively small estimated OR gene repertoire. However, in contrast to the kiwi and the kakapo, the snow petrel is a specialist diurnal forager (Ainley *et al*. 1984; Warham 1996) and it is plausible that its olfactory system has evolved to be highly sensitive to only a limited variety of odours. Based on our analysis, we predict that the OR gene repertoire of the zebra finch (*T. guttata*), whose genome sequence will soon become available, will be similar to that of the two passeriform genomes analysed here, approximately 200 OR genes.

We showed that OBR positively correlated with the estimated total number of OR genes, but not with the proportion of potentially functional OR genes, among the nine avian taxa examined. Thus, our results support the recent suggestion that the total number of OR genes, rather than the proportion of potentially functional OR genes, is a correlate of olfactory ability (Niimura & Nei 2006). To account for phylogeny, we based our analysis on Sibley & Ahlquist's (1991) comprehensive, but somewhat controversial, topology. This phylogeny was used because it provides branch lengths, and including these greatly increased the power of the statistical analysis. However, note that when more recently suggested avian phylogenies lacking branch lengths were applied (Cracraft *et al*. 2004), the correlation of estimated OR gene number with OBR was no longer significant (*r*=0.45, *n*=8, *p*=0.13, one-tailed). Hence, an investigation of the OR gene repertoires of more avian species is needed to verify whether OBR is indeed positively correlated with OR gene repertoire size. It has been suggested that the size of the olfactory epithelium indicates olfactory ability (see Issel-Tarver & Rine (1997) and references therein). However, we could not test the correlation between the surface of the olfactory epithelium and OR gene repertoire size, because very little information exists about the surface of the olfactory epithelium in birds (Hagelin 2006). This may be worthy of future exploration.

While it is likely that birds with both relatively large OBRs and OR gene repertoires have an excellent sense of smell, the opposite may not be true. Thus, birds with relatively small OBRs and relatively few OR genes do not necessarily lack a good sense of smell. For example, despite their relatively small OBR (9.7%; Bang & Cobb 1968), European starlings (*Sturnus vulgaris*) are able to detect and discriminate volatile compounds of plants (e.g. milfoil *Achillea millefolium*) incorporated into their nests during the breeding season (Clark & Mason 1987; Gwinner & Berger 2008). Similarly, blue tits (*C. caeruleus*) appear to use olfaction in their maintenance of an aromatic environment for nestlings (Petit *et al*. 2002; Hagelin 2006) and for predator detection (Amo *et al*. 2008). Thus, the relationship between olfactory acuity, olfactory anatomy and OR gene repertoire characteristics is not simple and requires further study.

As a large γ-c OR clade is present in all the avian genomes examined, the γ-c OR clade expansion may be a characteristic of all the bird genomes. Two lines of evidence indicate that the γ-c OR clade expansion did not occur before the divergence of the avian lineage. First, we used the same degenerate PCR primer pairs to amplify OR-coding sequences from Nile crocodile (*Crocodylus niloticus*) genomic DNA and no γ-c OR genes were identified (S. Steiger 2007, unpublished data). Second, we did not detect any group-γ-c OR genes in database searches of a draft reptilian genomic sequence (*Anolis* lizard, *Anolis carolinensis*: V. Kuryshev 2008, unpublished data). Because the large γ-c OR clade is also absent from mammalian genomes, we suggest that this OR clade is a basal, shared feature of class Aves.

The red jungle fowl γ-c OR clade members were predicted to be orthologous to human OR genes located next to major histocompatibility complex (MHC) class I gene clusters (International Chicken Genome Sequencing Consortium 2004). Interestingly, MHC-linked OR genes may play a role in mating preferences (Penn 2002). Chicken MHC genes have been localized on microchromosome 16 (Fillon *et al*. 1996). However, to our knowledge, OR genes have not been located nearby. Since the majority of chicken OR genes have not been assigned positions on the genome (see below), it remains to be seen whether avian γ-c OR clade members are in the proximity of MHC genes and/or are relevant for avian mate choice. Therefore, we suggest that future studies should investigate the functional significance of the apparently bird lineage-specific expanded γ-c OR clade.

The intermingling of the non-γ-c OR clade sequences of differing vertebrate taxa in the phylogenetic trees is compatible with the birth-and-death model of OR gene evolution, in which genes are created by repeated gene duplication and some genes later become non-functional (Nei 1969; for a review, see Nei & Rooney 2005). In addition, this pattern indicates that many of the OR gene lineage divergences pre-date the organism-level lineage divergences. Indeed, it is to be expected that a subset of the OR genes have evolutionarily conserved sequences and associated functions.

However, within the γ-c OR clade, sequences from the same, or closely related, species are very similar and therefore cluster together in phylogenetic trees. This clustering pattern may indicate that the γ-c OR clade may have arisen from independent expansion events or that the γ-c OR clade genes became homogenized by concerted evolution (Nei & Rooney 2005). Indeed, gene conversion has been shown to occur in closely related mammalian OR genes that are located together in a genomic cluster (Sharon *et al*. 1999). Interestingly, although the red jungle fowl γ-c OR genes have not yet been assigned to specific chromosomes (Build v. 2.1), BLAST searches have established that the 40 red jungle fowl γ-c OR genes identified by Niimura & Nei (2005) are located on 22 different contigs with a total length of 1691 kb. This represents only 0.14% of the total red jungle fowl genome, suggesting that the γ-c OR clade members may also be organized in clusters (data not shown). Such clustering promotes concerted evolution (Chen *et al*. 2007). Clearly, additional studies are needed to unravel both the molecular evolutionary history of the avian γ-c OR gene clade and its adaptive significance.

Available evidence suggests that OR genes with highly similar protein sequences bind structurally similar odorants (Malnic *et al*. 1999). If members of the large γ-c OR clade are functionally redundant, one would predict that loss-of-function mutations are not deleterious and, therefore, that a larger proportion of pseudogenes evolved in the γ-c OR clade than in the non-γ-c OR clades. However, the proportion of potentially functional OR genes does not differ significantly between the γ-c and non-γ-c OR clades, indicating that there is conservative or positive selection on genes forming the γ-c OR clade in all the avian genomes we examined.

In summary, our results support the growing body of evidence that the importance of the sense of smell for birds may have been greatly underestimated. In particular, the estimated OR gene repertoire sizes, and the proportion of OR genes that is potentially functional, contradict the general view that avian olfactory ability is poorly developed.

## Figures and Tables

**Figure 1:**
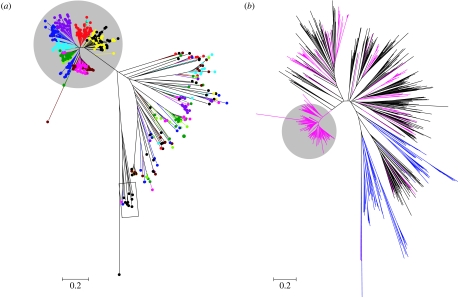
Unrooted neighbour-joining (NJ) phylogenetic trees derived from alignments of predicted vertebrate OR protein sequences (TM3–TM7). (*a*) The NJ phylogenetic tree of 483 predicted avian protein sequences derived from predicted functional OR genes from the canary (dark red, *S. canaria*, 44 sequences), the blue tit (pink, *C. caeruleus*, 55 sequences), the galah, (light green, *E. roseicapillus*, 19 sequences), the kakapo (dark green, *S. habroptilus*, 46 sequences), the black coucal (red, *C. grillii*, 53 sequences), the mallard (blue, *A. platyrhynchos*, 52 sequences), the red jungle fowl (yellow and black, *G. gallus*, 44 and 78 sequences), the snow petrel (cyan, *P. nivea*, 40 sequences) and the brown kiwi (purple, *A. australis*, 52 sequences). The red jungle fowl sequences that were obtained from Niimura & Nei (2005; *n*=78) are indicated by black circles, while the red jungle fowl sequences amplified in this study are indicated by yellow circles (*n*=44). Note that few group-α genes, indicated within the rectangle, were amplified using the primers and reaction conditions of this study. The large γ-c OR clade is shaded in grey. The scale bar indicates the number of amino acid substitutions per site. (*b*) Unrooted NJ trees generated from alignments of predicted vertebrate OR protein sequences: human (black lines, 388 sequences); zebrafish (blue lines, *Danio rerio*, 98 sequences); and avian (pink lines, 483 sequences). The predicted human and zebrafish OR protein sequences were obtained from Niimura & Nei (2005), while the avian OR sequences were from Niimura & Nei (2005; *G. gallus*, *n*=78) or this work. The γ-c OR clade is shaded in grey. The scale bar indicates the number of amino acid substitutions per site.

**Table 1:** Summary of data on relative olfactory bulb size (the OBR), the OR gene repertoire (the number of OR genes amplified, the number of pseudogenes amplified, the estimated percentage of potentially functional OR genes and the estimated total OR gene numbers) and ecological variables for the nine avian species.

common name	scientific name	order	OBR^a^	no. of OR genes amplified^b^	no. of pseudogenes amplified^b^	percentage of potentially functional OR genes	estimated total number of OR genes	ecological variables^c^ (habitat/diet/activity pattern)
canary	*Serinus canaria*	Passeriformes	6.0^d^	55 (19/36/0)	11 (1/10/0)	80	166	scrub/granivorous/diurnal
galah	*Eolophus roseicapillus*	Psittaciformes	8.0^d^^,^^e^	26 (17/9/0)	7 (3/4/0)	73.1	107	grassland and agricultural areas/herbivorous/diurnal
blue tit	*Cyanistes caeruleus*	Passeriformes	9.7^d^	62 (18/43/1)	7 (6/1/0)	88.7	218	forest/omnivorous/diurnal
red jungle fowl	*Gallus gallus*	Galliformes	14.2^d^	46 (11/35/0)	2 (0/2/0)	95.7	638	forest/omnivorous/diurnal
mallard	*Anas platyrhynchos*	Anseriformes	19.4^d^	61 (14/47/1)	9 (0/9/0)	85.2	430	wetlands/omnivorous/diurnal
black coucal	*Centropus grillii*	Cuculiformes	19.5^d^^,^^e^	61 (9/52/0)	8 (0/8/0)	86.9	—	grassland/insectivorous/diurnal
kakapo	*Strigops habroptilus*	Psittaciformes	30.2^f^	56 (21/35/0)	10 (2/8/0)	82.1	667	forest/herbivorous/nocturnal
brown kiwi	*Apteryx australis*	Apterygiformes	34.0^d^	68 (29/39/0)	16 (8/8/0)	76.5	600	forest and scrub/insectivorous/nocturnal
snow petrel	*Pagodroma nivea*	Procellariiformes	37.0^d^	47 (21/26/0)	7 (3/4/0)	85.1	212	marine/planktivorous and piscivorous/diurnal

a The OBR is the ratio of the greatest diameter of the olfactory bulb relative to the greatest diameter of the cerebral hemisphere, expressed as a percentage.

b Numbers in brackets refer to non-γ-c OR genes, γ-c OR genes and group-α OR genes, respectively.

c From Cramp & Perrins (1992), del Hoyo *et al*. (1992, 1997, 2007), Elliot *et al*. (1994) and Bonadonna & Bretagnolle (2002).

d From Bang & Cobb (1968).

e Mean OBR of this order.

f From Hagelin (2004).

## Supplementary Material

Methodological and sequence details *Supplementary information*. The OR sequences generated in this study were deposited to GenBank (http://www.ncbi.nih.gov/Genbank/) with accession numbers EF426863–EF427345 (see table S1 of the electronic supplementary material)
