# Supplementary material for: Avian olfactory receptor gene repertoires: evidence for a well-developed sense of smell in birds?
Source: Proc Biol Sci. 2008 Jul 15;275(1649):2309–17. doi: 10.1098/rspb.2008.0607 (PMC2495045; doi:10.1098/rspb.2008.0607)
Supplement: Supplementary file 1 [file rspb20080607s02.doc]

**Electronic SupplementaRY MATERIAL (STeiger *et al.*)**

Methodological and sequence details

**Figure S1.** Phylogeny used for calculating phylogenetic independent contrasts of (a) the number of OR genes on olfactory bulb size ratio for 8 avian species and (b) the proportion of potentially functional OR genes on olfactory bulb size ratio for 9 avian species. The topology of the tree and branch lengths were obtained by using genetic distances derived from DNA-DNA hybridization studies (Sibley & Ahlquist 1991). Nodes are indicated with numbers.

**Figure S2**. Phylogenetic independent contrasts. **(**a) The relationship between phylogenetic independent contrasts of the number of OR genes on olfactory bulb size ratio for 8 avian species. Numbers refer to the nodes in Fig. S1a. (b) The relationship between phylogenetic independent contrasts of the proportion of potentially functional OR genes on olfactory bulb size ratio for 9 avian species. Numbers refer to the nodes in Fig. S1b.

**Fig. S1.**

(a)

(b)

**Fig. S2.**

**TABLE S1.** Summary of avian olfactory receptor genes.

| **Common name** | **OR gene** | **Potentially functional / Pseudogene** | **Copies** | **Primer pair** | **GenBank accession number** |
| --- | --- | --- | --- | --- | --- |
| Canary | ScOR1 | Functional | 1 | ICKPLHY / NPFIYS(F/L) | EF426925 |
| Canary | ScOR2 | Pseudogene | 1 | ICKPLHY / NPFIYS(F/L) | EF426926 |
| Canary | ScOR3 | Pseudogene | 1 | ICKPLHY / NPFIYS(F/L) | EF426927 |
| Canary | ScOR4 | Functional | 4 | ICKPLHY / NPFIYS(F/L) | EF426928 |
| Canary | ScOR5 | Functional | 1 | ICKPLHY / NPFIYS(F/L) | EF426929 |
| Canary | ScOR6 | Pseudogene | 2 | ICKPLHY / NPFIYS(F/L) | EF426930 |
| Canary | ScOR7 | Pseudogene | 2 | ICKPLHY / NPFIYS(F/L) | EF426931 |
| Canary | ScOR8 | Functional | 1 | ICKPLHY / NPFIYS(F/L) | EF426932 |
| Canary | ScOR9 | Pseudogene | 1 | ICKPLHY / NPFIYS(F/L) | EF426933 |
| Canary | ScOR10 | Functional | 2 | ICKPLHY / NPFIYS(F/L) | EF426934 |
| Canary | ScOR11 | Functional | 3 | ICKPLHY / NPFIYS(F/L) | EF426935 |
| Canary | ScOR12 | Functional | 2 | ICKPLHY / NPFIYS(F/L) | EF426936 |
| Canary | ScOR13 | Pseudogene | 2 | ICKPLHY / NPFIYS(F/L) | EF426937 |
| Canary | ScOR14 | Functional | 1 | ICKPLHY / NPFIYS(F/L) | EF426938 |
| Canary | ScOR15 | Functional | 3 | ICKPLHY / NPFIYS(F/L) | EF426939 |
| Canary | ScOR16 | Functional | 1 | ICKPLHY / NPFIYS(F/L) | EF426940 |
| Canary | ScOR17 | Functional | 1 | ICKPLHY / NPFIYS(F/L) | EF426941 |
| Canary | ScOR18 | Functional | 2 | VAICKPLHY / NPFIYS(F/L) | EF426942 |
| Canary | ScOR19 | Functional | 1 | VAICKPLHY / NPFIYS(F/L) | EF426943 |
| Canary | ScOR20 | Functional | 1 | VAICKPLHY / NPFIYS(F/L) | EF426944 |
| Canary | ScOR21 | Functional | 1 | VAICKPLHY / NPFIYS(F/L) | EF426945 |
| Canary | ScOR22 | Functional | 3 | VAICKPLHY / NPFIYS(F/L) | EF426946 |
| Canary | ScOR23 | Functional | 1 | VAICKPLHY / NPFIYS(F/L) | EF426947 |
| Canary | ScOR24 | Functional | 2 | VAICKPLHY / NPFIYS(F/L) | EF426948 |
| Canary | ScOR25 | Functional | 1 | VAICKPLHY / NPFIYS(F/L) | EF426949 |
| Canary | ScOR26 | Functional | 1 | VAICKPLHY / NPFIYS(F/L) | EF426950 |
| Canary | ScOR27 | Functional | 1 | VAICKPLHY / NPFIYS(F/L) | EF426951 |
| Canary | ScOR28 | Functional | 1 | VAICKPLHY / NPFIYS(F/L) | EF426952 |
| Canary | ScOR29 | Pseudogene | 1 | ICKPLHY / NPFIYS(F/L) | EF426953 |
| Canary | ScOR30 | Pseudogene | 1 | ICKPLHY / NPFIYS(F/L) | EF426954 |
| Canary | ScOR31 | Functional | 1 | VAICKPLHY / NPFIYS(F/L) | EF426955 |
| Canary | ScOR32 | Functional | 5 | MAYDRY / PMLNPLIY | EF426956 |
| Canary | ScOR33 | Functional | 2 | MAYDRY / PMLNPLIY | EF426957 |
| Canary | ScOR34 | Functional | 1 | MAYDRY / PMLNPLIY | EF426958 |
| Canary | ScOR35 | Functional | 1 | MAYDRY / PMLNPLIY | EF426959 |
| Canary | ScOR36 | Functional | 9 | MAYDRY / PMLNPLIY | EF426960 |
| Canary | ScOR37 | Functional | 4 | MAYDRY / NPFIYS(F/L) | EF426961 |
| Canary | ScOR38 | Functional | 2 | MAYDRY / NPFIYS(F/L) | EF426962 |
| Canary | ScOR39 | Pseudogene | 1 | MAYDRY / NPFIYS(F/L) | EF426963 |
| Canary | ScOR40 | Pseudogene | 1 | MAYDRY / NPFIYS(F/L) | EF426964 |
| Canary | ScOR41 | Functional | 1 | MAYDRY / NPFIYS(F/L) | EF426965 |
| Canary | ScOR42 | Functional | 3 | MAYDRY / NPFIYS(F/L) | EF426966 |
| Canary | ScOR43 | Pseudogene | 1 | MAYDRY / PM(L/F)NP | EF426967 |
| Canary | ScOR44 | Functional | 1 | MAYDRY / PM(L/F)NP | EF426968 |
| Canary | ScOR45 | Functional | 1 | MAYDRY / PM(L/F)NP | EF426969 |
| Canary | ScOR46 | Functional | 1 | MAYDRY / PM(L/F)NP | EF426970 |
| Canary | ScOR47 | Functional | 1 | MAYDRY / PM(L/F)NP | EF426971 |
| Canary | ScOR48 | Functional | 4 | AMAYDRY / PMLNPLIY | EF426972 |
| Canary | ScOR49 | Functional | 1 | AMAYDRY / PMLNPLIY | EF426973 |
| Canary | ScOR50 | Functional | 1 | AMAYDRY / PMLNPLIY | EF426974 |
| Canary | ScOR51 | Functional | 2 | AMAYDRY / PMLNPLIY | EF426975 |
| Canary | ScOR52 | Functional | 1 | AMAYDRY / NPFIYS(F/L) | EF426976 |
| Canary | ScOR53 | Functional | 2 | AMAYDRY / PM(L/F)NP | EF426977 |
| Canary | ScOR54 | Functional | 1 | MAYDRY(V/L)AIC / PMLNPLIY | EF426978 |
| Canary | ScOR55 | Functional | 7 | MAYDRY(V/L)AIC / PMLNPLIY | EF426979 |
| Galah | ErOR1 | Functional | 1 | ICKPLHY / NPFIYS(F/L) | EF427041 |
| Galah | ErOR2 | Pseudogene | 3 | ICKPLHY / NPFIYS(F/L) | EF427042 |
| Galah | ErOR3 | Pseudogene | 1 | ICKPLHY / NPFIYS(F/L) | EF427043 |
| Galah | ErOR4 | Pseudogene | 1 | ICKPLHY / NPFIYS(F/L) | EF427044 |
| Galah | ErOR5 | Functional | 2 | ICKPLHY / NPFIYS(F/L) | EF427045 |
| Galah | ErOR6 | Functional | 1 | ICKPLHY / NPFIYS(F/L) | EF427046 |
| Galah | ErOR7 | Pseudogene | 1 | ICKPLHY / NPFIYS(F/L) | EF427047 |
| Galah | ErOR8 | Functional | 1 | ICKPLHY / NPFIYS(F/L) | EF427048 |
| Galah | ErOR9 | Functional | 1 | VAICKPLHY / NPFIYS(F/L) | EF427049 |
| Galah | ErOR10 | Functional | 2 | MAYDRY / PM(L/F)NP | EF427050 |
| Galah | ErOR11 | Functional | 10 | MAYDRY / PM(L/F)NP | EF427051 |
| Galah | ErOR12 | Functional | 2 | MAYDRY / PM(L/F)NP | EF427052 |
| Galah | ErOR13 | Functional | 1 | MAYDRY / NPFIYS(F/L) | EF427053 |
| Galah | ErOR14 | Functional | 1 | MAYDRY / NPFIYS(F/L) | EF427054 |
| Galah | ErOR15 | Functional | 3 | MAYDRY / PMLNPLIY | EF427055 |
| Galah | ErOR16 | Functional | 1 | MAYDRY / PMLNPLIY | EF427056 |
| Galah | ErOR17 | Functional | 3 | AMAYDRY / NPFIYS(F/L) | EF427057 |
| Galah | ErOR18 | Functional | 2 | AMAYDRY / PM(L/F)NP | EF427058 |
| Galah | ErOR19 | Pseudogene | 1 | AMAYDRY / NPFIYS(F/L) | EF427059 |
| Galah | ErOR20 | Functional | 2 | AMAYDRY / NPFIYS(F/L) | EF427060 |
| Galah | ErOR21 | Functional | 1 | MAYDRY(V/L)AIC / NPFIYS(F/L) | EF427061 |
| Galah | ErOR22 | Functional | 1 | AMAYDRY / PMLNPLIY | EF427062 |
| Galah | ErOR23 | Functional | 1 | MAYDRY / PM(L/F)NP | EF427063 |
| Galah | ErOR24 | Pseudogene | 1 | MAYDRY(V/L)AIC / PMLNPLIY | EF427064 |
| Galah | ErOR25 | Functional | 1 | MAYDRY(V/L)AIC / PM(L/F)NP | EF427065 |
| Galah | ErOR26 | Pseudogene | 2 | MAYDRY / PM(L/F)NP | EF427066 |
| Blue tit | CcOR1 | Functional | 2 | VAICKPLHY / NPFIYS(F/L) | EF426863 |
| Blue tit | CcOR2 | Functional | 7 | VAICKPLHY / NPFIYS(F/L) | EF426864 |
| Blue tit | CcOR3 | Functional | 4 | VAICKPLHY / NPFIYS(F/L) | EF426865 |
| Blue tit | CcOR4 | Functional | 1 | VAICKPLHY / NPFIYS(F/L) | EF426866 |
| Blue tit | CcOR5 | Functional | 1 | VAICKPLHY / NPFIYS(F/L) | EF426867 |
| Blue tit | CcOR6 | Functional | 2 | VAICKPLHY / NPFIYS(F/L) | EF426868 |
| Blue tit | CcOR7 | Functional | 2 | VAICKPLHY / NPFIYS(F/L) | EF426869 |
| Blue tit | CcOR8 | Pseudogene | 1 | VAICKPLHY / NPFIYS(F/L) | EF426870 |
| Blue tit | CcOR9 | Functional | 1 | VAICKPLHY / NPFIYS(F/L) | EF426871 |
| Blue tit | CcOR10 | Pseudogene | 1 | VAICKPLHY / NPFIYS(F/L) | EF426872 |
| Blue tit | CcOR11 | Functional | 2 | VAICKPLHY / NPFIYS(F/L) | EF426873 |
| Blue tit | CcOR12 | Functional | 1 | VAICKPLHY / NPFIYS(F/L) | EF426874 |
| Blue tit | CcOR13 | Functional | 2 | ICKPLHY / NPFIYS(F/L) | EF426875 |
| Blue tit | CcOR14 | Functional | 1 | ICKPLHY / NPFIYS(F/L) | EF426876 |
| Blue tit | CcOR15 | Functional | 1 | ICKPLHY / NPFIYS(F/L) | EF426877 |
| Blue tit | CcOR16 | Functional | 2 | ICKPLHY / NPFIYS(F/L) | EF426878 |
| Blue tit | CcOR17 | Functional | 1 | ICKPLHY / NPFIYS(F/L) | EF426879 |
| Blue tit | CcOR18 | Functional | 2 | ICKPLHY / NPFIYS(F/L) | EF426880 |
| Blue tit | CcOR19 | Functional | 1 | ICKPLHY / NPFIYS(F/L) | EF426881 |
| Blue tit | CcOR20 | Functional | 1 | ICKPLHY / NPFIYS(F/L) | EF426882 |
| Blue tit | CcOR21 | Functional | 2 | ICKPLHY / NPFIYS(F/L) | EF426883 |
| Blue tit | CcOR22 | Functional | 1 | ICKPLHY / NPFIYS(F/L) | EF426884 |
| Blue tit | CcOR23 | Functional | 1 | ICKPLHY / NPFIYS(F/L) | EF426885 |
| Blue tit | CcOR24 | Functional | 1 | ICKPLHY / NPFIYS(F/L) | EF426886 |
| Blue tit | CcOR25 | Functional | 1 | ICKPLHY / NPFIYS(F/L) | EF426887 |
| Blue tit | CcOR26 | Functional | 1 | ICKPLHY / NPFIYS(F/L) | EF426888 |
| Blue tit | CcOR27 | Functional | 1 | ICKPLHY / NPFIYS(F/L) | EF426889 |
| Blue tit | CcOR28 | Functional | 1 | ICKPLHY / NPFIYS(F/L) | EF426890 |
| Blue tit | CcOR29 | Functional | 1 | ICKPLHY / NPFIYS(F/L) | EF426891 |
| Blue tit | CcOR30 | Functional | 1 | ICKPLHY / NPFIYS(F/L) | EF426892 |
| Blue tit | CcOR31 | Functional | 1 | ICKPLHY / NPFIYS(F/L) | EF426893 |
| Blue tit | CcOR32 | Functional | 1 | ICKPLHY / NPFIYS(F/L) | EF426894 |
| Blue tit | CcOR33 | Functional | 1 | ICKPLHY / NPFIYS(F/L) | EF426895 |
| Blue tit | CcOR34 | Functional | 2 | ICKPLHY / NPFIYS(F/L) | EF426896 |
| Blue tit | CcOR35 | Functional | 1 | ICKPLHY / NPFIYS(F/L) | EF426897 |
| Blue tit | CcOR36 | Functional | 1 | ICKPLHY / NPFIYS(F/L) | EF426898 |
| Blue tit | CcOR37 | Functional | 1 | ICKPLHY / NPFIYS(F/L) | EF426899 |
| Blue tit | CcOR38 | Functional | 1 | ICKPLHY / NPFIYS(F/L) | EF426900 |
| Blue tit | CcOR39 | Functional | 1 | VAICKPLHY / NPFIYS(F/L) | EF426901 |
| Blue tit | CcOR40 | Functional | 2 | VAICKPLHY / NPFIYS(F/L) | EF426902 |
| Blue tit | CcOR41 | Functional | 1 | VAICKPLHY / NPFIYS(F/L) | EF426903 |
| Blue tit | CcOR42 | Pseudogene | 1 | MAYDRY / PMLNPLIY | EF426904 |
| Blue tit | CcOR43 | Pseudogene | 1 | MAYDRY / PMLNPLIY | EF426905 |
| Blue tit | CcOR44 | Functional | 4 | MAYDRY / PM(L/F)NP | EF426906 |
| Blue tit | CcOR45 | Pseudogene | 8 | MAYDRY / PM(L/F)NP | EF426907 |
| Blue tit | CcOR46 | Pseudogene | 3 | MAYDRY / PM(L/F)NP | EF426908 |
| Blue tit | CcOR47 | Functional | 1 | AMAYDRY / NPFIYS(F/L) | EF426909 |
| Blue tit | CcOR48 | Pseudogene | 2 | AMAYDRY / NPFIYS(F/L) | EF426910 |
| Blue tit | CcOR49 | Functional | 1 | AMAYDRY / PM(L/F)NP | EF426911 |
| Blue tit | CcOR50 | Functional | 1 | MAYDRY(V/L)AIC / NPFIYS(F/L) | EF426912 |
| Blue tit | CcOR51 | Functional | 2 | MAYDRY(V/L)AIC / NPFIYS(F/L) | EF426913 |
| Blue tit | CcOR52 | Functional | 1 | MAYDRY(V/L)AIC / NPFIYS(F/L) | EF426914 |
| Blue tit | CcOR53 | Functional | 2 | MAYDRY(V/L)AIC / NPFIYS(F/L) | EF426915 |
| Blue tit | CcOR54 | Functional | 1 | MAYDRY(V/L)AIC / NPFIYS(F/L) | EF426916 |
| Blue tit | CcOR55 | Functional | 1 | AMAYDRY / NPFIYS(F/L) | EF426917 |
| Blue tit | CcOR56 | Functional | 2 | AMAYDRY / NPFIYS(F/L) | EF426918 |
| Blue tit | CcOR57 | Functional | 1 | AMAYDRY / PM(L/F)NP | EF426919 |
| Blue tit | CcOR58 | Functional | 2 | MAYDRY(V/L)AIC / NPFIYS(F/L) | EF426920 |
| Blue tit | CcOR59 | Functional | 1 | MAYDRY(V/L)AIC / NPFIYS(F/L) | EF426921 |
| Blue tit | CcOR60 | Functional | 2 | MAYDRY(V/L)AIC / NPFIYS(F/L) | EF426922 |
| Blue tit | CcOR61 | Functional | 1 | MAYDRY(V/L)AIC / NPFIYS(F/L) | EF426923 |
| Blue tit | CcOR62 | Functional | 1 | MAYDRY(V/L)AIC / PM(L/F)NP | EF426924 |
| Jungle fowl | GgOR1 | Functional | 1 | ICKPLHY / NPFIYS(F/L) | EF427067 |
| Jungle fowl | GgOR2 | Functional | 3 | ICKPLHY / NPFIYS(F/L) | EF427068 |
| Jungle fowl | GgOR3 | Functional | 1 | ICKPLHY / NPFIYS(F/L) | EF427069 |
| Jungle fowl | GgOR4 | Functional | 1 | ICKPLHY / NPFIYS(F/L) | EF427070 |
| Jungle fowl | GgOR5 | Functional | 4 | ICKPLHY / NPFIYS(F/L) | EF427071 |
| Jungle fowl | GgOR6 | Functional | 4 | ICKPLHY / NPFIYS(F/L) | EF427072 |
| Jungle fowl | GgOR7 | Functional | 2 | ICKPLHY / NPFIYS(F/L) | EF427073 |
| Jungle fowl | GgOR8 | Functional | 1 | ICKPLHY / NPFIYS(F/L) | EF427074 |
| Jungle fowl | GgOR9 | Functional | 1 | ICKPLHY / NPFIYS(F/L) | EF427075 |
| Jungle fowl | GgOR10 | Functional | 1 | ICKPLHY / NPFIYS(F/L) | EF427076 |
| Jungle fowl | GgOR11 | Functional | 1 | ICKPLHY / NPFIYS(F/L) | EF427077 |
| Jungle fowl | GgOR12 | Functional | 1 | ICKPLHY / NPFIYS(F/L) | EF427078 |
| Jungle fowl | GgOR13 | Functional | 3 | ICKPLHY / NPFIYS(F/L) | EF427079 |
| Jungle fowl | GgOR14 | Functional | 1 | VAICKPLHY / NPFIYS(F/L) | EF427080 |
| Jungle fowl | GgOR15 | Functional | 1 | VAICKPLHY / NPFIYS(F/L) | EF427081 |
| Jungle fowl | GgOR16 | Functional | 1 | VAICKPLHY / NPFIYS(F/L) | EF427082 |
| Jungle fowl | GgOR17 | Functional | 1 | VAICKPLHY / NPFIYS(F/L) | EF427083 |
| Jungle fowl | GgOR18 | Functional | 1 | VAICKPLHY / NPFIYS(F/L) | EF427084 |
| Jungle fowl | GgOR19 | Functional | 1 | VAICKPLHY / NPFIYS(F/L) | EF427085 |
| Jungle fowl | GgOR20 | Functional | 1 | VAICKPLHY / NPFIYS(F/L) | EF427086 |
| Jungle fowl | GgOR21 | Functional | 2 | VAICKPLHY / NPFIYS(F/L) | EF427087 |
| Jungle fowl | GgOR22 | Functional | 3 | VAICKPLHY / NPFIYS(F/L) | EF427088 |
| Jungle fowl | GgOR23 | Pseudogene | 1 | VAICKPLHY / NPFIYS(F/L) | EF427089 |
| Jungle fowl | GgOR24 | Functional | 1 | VAICKPLHY / NPFIYS(F/L) | EF427090 |
| Jungle fowl | GgOR25 | Functional | 2 | VAICKPLHY / NPFIYS(F/L) | EF427091 |
| Jungle fowl | GgOR26 | Functional | 1 | VAICKPLHY / NPFIYS(F/L) | EF427092 |
| Jungle fowl | GgOR27 | Functional | 1 | VAICKPLHY / NPFIYS(F/L) | EF427093 |
| Jungle fowl | GgOR28 | Functional | 1 | VAICKPLHY / NPFIYS(F/L) | EF427094 |
| Jungle fowl | GgOR29 | Functional | 1 | VAICKPLHY / NPFIYS(F/L) | EF427095 |
| Jungle fowl | GgOR30 | Pseudogene | 1 | VAICKPLHY / NPFIYS(F/L) | EF427096 |
| Jungle fowl | GgOR31 | Functional | 1 | VAICKPLHY / NPFIYS(F/L) | EF427097 |
| Jungle fowl | GgOR32 | Functional | 1 | VAICKPLHY / NPFIYS(F/L) | EF427098 |
| Jungle fowl | GgOR33 | Functional | 4 | MAYDRY / PMLNPLIY | EF427099 |
| Jungle fowl | GgOR34 | Functional | 31 | AMAYDRY / NPFIYS(F/L) | EF427100 |
| Jungle fowl | GgOR35 | Functional | 13 | AMAYDRY / PM(L/F)NP | EF427101 |
| Jungle fowl | GgOR36 | Functional | 6 | AMAYDRY / PMLNPLIY | EF427102 |
| Jungle fowl | GgOR37 | Functional | 2 | MAYDRY(V/L)AIC / NPFIYS(F/L) | EF427103 |
| Jungle fowl | GgOR38 | Functional | 1 | MAYDRY(V/L)AIC / NPFIYS(F/L) | EF427104 |
| Jungle fowl | GgOR39 | Functional | 1 | MAYDRY(V/L)AIC / NPFIYS(F/L) | EF427105 |
| Jungle fowl | GgOR40 | Functional | 1 | MAYDRY(V/L)AIC / NPFIYS(F/L) | EF427106 |
| Jungle fowl | GgOR41 | Functional | 5 | MAYDRY / PMLNPLIY | EF427107 |
| Jungle fowl | GgOR42 | Functional | 3 | MAYDRY / PMLNPLIY | EF427108 |
| Jungle fowl | GgOR43 | Functional | 1 | AMAYDRY / PMLNPLIY | EF427109 |
| Jungle fowl | GgOR44 | Functional | 1 | MAYDRY(V/L)AIC / NPFIYS(F/L) | EF427110 |
| Jungle fowl | GgOR45 | Functional | 6 | AMAYDRY / PM(L/F)NP | EF427111 |
| Jungle fowl | GgOR46 | Functional | 4 | MAYDRY / PMLNPLIY | EF427112 |
| Mallard | ApOR1 | Functional | 4 | ICKPLHY / NPFIYS(F/L) | EF427237 |
| Mallard | ApOR2 | Pseudogene | 2 | ICKPLHY / NPFIYS(F/L) | EF427238 |
| Mallard | ApOR3 | Pseudogene | 1 | ICKPLHY / NPFIYS(F/L) | EF427239 |
| Mallard | ApOR4 | Functional | 1 | ICKPLHY / NPFIYS(F/L) | EF427240 |
| Mallard | ApOR5 | Pseudogene | 1 | ICKPLHY / NPFIYS(F/L) | EF427241 |
| Mallard | ApOR6 | Functional | 1 | ICKPLHY / NPFIYS(F/L) | EF427242 |
| Mallard | ApOR7 | Functional | 1 | ICKPLHY / NPFIYS(F/L) | EF427243 |
| Mallard | ApOR8 | Functional | 1 | ICKPLHY / NPFIYS(F/L) | EF427244 |
| Mallard | ApOR9 | Functional | 2 | ICKPLHY / NPFIYS(F/L) | EF427245 |
| Mallard | ApOR10 | Functional | 1 | ICKPLHY / NPFIYS(F/L) | EF427246 |
| Mallard | ApOR11 | Functional | 1 | ICKPLHY / NPFIYS(F/L) | EF427247 |
| Mallard | ApOR12 | Functional | 1 | ICKPLHY / NPFIYS(F/L) | EF427248 |
| Mallard | ApOR13 | Functional | 1 | ICKPLHY / NPFIYS(F/L) | EF427249 |
| Mallard | ApOR14 | Functional | 1 | ICKPLHY / NPFIYS(F/L) | EF427250 |
| Mallard | ApOR15 | Functional | 1 | ICKPLHY / NPFIYS(F/L) | EF427251 |
| Mallard | ApOR16 | Functional | 2 | ICKPLHY / NPFIYS(F/L) | EF427252 |
| Mallard | ApOR17 | Functional | 2 | VAICKPLHY / NPFIYS(F/L) | EF427253 |
| Mallard | ApOR18 | Functional | 1 | VAICKPLHY / NPFIYS(F/L) | EF427254 |
| Mallard | ApOR19 | Functional | 3 | VAICKPLHY / NPFIYS(F/L) | EF427255 |
| Mallard | ApOR20 | Functional | 1 | VAICKPLHY / NPFIYS(F/L) | EF427256 |
| Mallard | ApOR21 | Pseudogene | 1 | VAICKPLHY / NPFIYS(F/L) | EF427257 |
| Mallard | ApOR22 | Pseudogene | 1 | VAICKPLHY / NPFIYS(F/L) | EF427258 |
| Mallard | ApOR23 | Functional | 2 | VAICKPLHY / NPFIYS(F/L) | EF427259 |
| Mallard | ApOR24 | Functional | 1 | VAICKPLHY / NPFIYS(F/L) | EF427260 |
| Mallard | ApOR25 | Functional | 1 | VAICKPLHY / NPFIYS(F/L) | EF427261 |
| Mallard | ApOR26 | Functional | 2 | VAICKPLHY / NPFIYS(F/L) | EF427262 |
| Mallard | ApOR27 | Functional | 1 | VAICKPLHY / NPFIYS(F/L) | EF427263 |
| Mallard | ApOR28 | Functional | 1 | VAICKPLHY / NPFIYS(F/L) | EF427264 |
| Mallard | ApOR29 | Functional | 1 | VAICKPLHY / NPFIYS(F/L) | EF427265 |
| Mallard | ApOR30 | Functional | 1 | VAICKPLHY / NPFIYS(F/L) | EF427266 |
| Mallard | ApOR31 | Functional | 1 | ICKPLHY / NPFIYS(F/L) | EF427267 |
| Mallard | ApOR32 | Functional | 1 | ICKPLHY / NPFIYS(F/L) | EF427268 |
| Mallard | ApOR33 | Functional | 1 | VAICKPLHY / NPFIYS(F/L) | EF427269 |
| Mallard | ApOR34 | Pseudogene | 1 | VAICKPLHY / NPFIYS(F/L) | EF427270 |
| Mallard | ApOR35 | Functional | 5 | AMAYDRY / NPFIYS(F/L) | EF427271 |
| Mallard | ApOR36 | Functional | 1 | MAYDRY / NPFIYS(F/L) | EF427272 |
| Mallard | ApOR37 | Pseudogene | 1 | MAYDRY / NPFIYS(F/L) | EF427273 |
| Mallard | ApOR38 | Functional | 1 | MAYDRY / NPFIYS(F/L) | EF427274 |
| Mallard | ApOR39 | Functional | 1 | MAYDRY / NPFIYS(F/L) | EF427275 |
| Mallard | ApOR40 | Functional | 1 | MAYDRY / NPFIYS(F/L) | EF427276 |
| Mallard | ApOR41 | Functional | 2 | MAYDRY / NPFIYS(F/L) | EF427277 |
| Mallard | ApOR42 | Functional | 4 | MAYDRY / PM(L/F)NP | EF427278 |
| Mallard | ApOR43 | Functional | 4 | AMAYDRY / PMLNPLIY | EF427279 |
| Mallard | ApOR44 | Functional | 1 | AMAYDRY / PMLNPLIY | EF427280 |
| Mallard | ApOR45 | Functional | 15 | AMAYDRY / PMLNPLIY | EF427281 |
| Mallard | ApOR46 | Functional | 5 | AMAYDRY / PMLNPLIY | EF427282 |
| Mallard | ApOR47 | Functional | 3 | AMAYDRY / PMLNPLIY | EF427283 |
| Mallard | ApOR48 | Functional | 2 | AMAYDRY / PMLNPLIY | EF427284 |
| Mallard | ApOR49 | Pseudogene | 1 | AMAYDRY / NPFIYS(F/L) | EF427285 |
| Mallard | ApOR50 | Functional | 1 | AMAYDRY / NPFIYS(F/L) | EF427286 |
| Mallard | ApOR51 | Functional | 1 | AMAYDRY / NPFIYS(F/L) | EF427287 |
| Mallard | ApOR52 | Functional | 1 | AMAYDRY / PM(L/F)NP | EF427288 |
| Mallard | ApOR53 | Functional | 1 | MAYDRY(V/L)AIC / NPFIYS(F/L) | EF427289 |
| Mallard | ApOR54 | Functional | 5 | AMAYDRY / NPFIYS(F/L) | EF427290 |
| Mallard | ApOR55 | Functional | 1 | MAYDRY / PMLNPLIY | EF427291 |
| Mallard | ApOR56 | Functional | 1 | MAYDRY(V/L)AIC / NPFIYS(F/L) | EF427292 |
| Mallard | ApOR57 | Pseudogene | 2 | AMAYDRY / NPFIYS(F/L) | EF427293 |
| Mallard | ApOR58 | Functional | 1 | MAYDRY / NPFIYS(F/L) | EF427294 |
| Mallard | ApOR59 | Functional | 1 | MAYDRY / NPFIYS(F/L) | EF427295 |
| Mallard | ApOR60 | Functional | 5 | MAYDRY / NPFIYS(F/L) | EF427296 |
| Mallard | ApOR61 | Functional | 1 | AMAYDRY / PMLNPLIY | EF427297 |
| Black coucal | CgOR1 | Functional | 1 | ICKPLHY / NPFIYS(F/L) | EF426980 |
| Black coucal | CgOR2 | Functional | 2 | ICKPLHY / NPFIYS(F/L) | EF426981 |
| Black coucal | CgOR3 | Pseudogene | 1 | ICKPLHY / NPFIYS(F/L) | EF426982 |
| Black coucal | CgOR4 | Functional | 1 | ICKPLHY / NPFIYS(F/L) | EF426983 |
| Black coucal | CgOR5 | Functional | 1 | ICKPLHY / NPFIYS(F/L) | EF426984 |
| Black coucal | CgOR6 | Pseudogene | 1 | ICKPLHY / NPFIYS(F/L) | EF426985 |
| Black coucal | CgOR7 | Functional | 1 | ICKPLHY / NPFIYS(F/L) | EF426986 |
| Black coucal | CgOR8 | Functional | 2 | ICKPLHY / NPFIYS(F/L) | EF426987 |
| Black coucal | CgOR9 | Functional | 1 | ICKPLHY / NPFIYS(F/L) | EF426988 |
| Black coucal | CgOR10 | Functional | 1 | ICKPLHY / NPFIYS(F/L) | EF426989 |
| Black coucal | CgOR11 | Functional | 1 | ICKPLHY / NPFIYS(F/L) | EF426990 |
| Black coucal | CgOR12 | Functional | 2 | ICKPLHY / NPFIYS(F/L) | EF426991 |
| Black coucal | CgOR13 | Functional | 1 | ICKPLHY / NPFIYS(F/L) | EF426992 |
| Black coucal | CgOR14 | Pseudogene | 1 | ICKPLHY / NPFIYS(F/L) | EF426993 |
| Black coucal | CgOR15 | Functional | 1 | ICKPLHY / NPFIYS(F/L) | EF426994 |
| Black coucal | CgOR16 | Functional | 2 | ICKPLHY / NPFIYS(F/L) | EF426995 |
| Black coucal | CgOR17 | Pseudogene | 1 | ICKPLHY / NPFIYS(F/L) | EF426996 |
| Black coucal | CgOR18 | Functional | 1 | ICKPLHY / NPFIYS(F/L) | EF426997 |
| Black coucal | CgOR19 | Functional | 1 | ICKPLHY / NPFIYS(F/L) | EF426998 |
| Black coucal | CgOR20 | Functional | 1 | ICKPLHY / NPFIYS(F/L) | EF426999 |
| Black coucal | CgOR21 | Functional | 1 | ICKPLHY / NPFIYS(F/L) | EF427000 |
| Black coucal | CgOR22 | Functional | 1 | ICKPLHY / NPFIYS(F/L) | EF427001 |
| Black coucal | CgOR23 | Functional | 1 | ICKPLHY / NPFIYS(F/L) | EF427002 |
| Black coucal | CgOR24 | Functional | 1 | ICKPLHY / NPFIYS(F/L) | EF427003 |
| Black coucal | CgOR25 | Functional | 1 | ICKPLHY / NPFIYS(F/L) | EF427004 |
| Black coucal | CgOR26 | Functional | 1 | ICKPLHY / NPFIYS(F/L) | EF427005 |
| Black coucal | CgOR27 | Functional | 1 | ICKPLHY / NPFIYS(F/L) | EF427006 |
| Black coucal | CgOR28 | Functional | 1 | ICKPLHY / NPFIYS(F/L) | EF427007 |
| Black coucal | CgOR29 | Functional | 1 | ICKPLHY / NPFIYS(F/L) | EF427008 |
| Black coucal | CgOR30 | Functional | 1 | ICKPLHY / NPFIYS(F/L) | EF427009 |
| Black coucal | CgOR31 | Functional | 1 | ICKPLHY / NPFIYS(F/L) | EF427010 |
| Black coucal | CgOR32 | Functional | 1 | ICKPLHY / NPFIYS(F/L) | EF427011 |
| Black coucal | CgOR33 | Functional | 1 | ICKPLHY / NPFIYS(F/L) | EF427012 |
| Black coucal | CgOR34 | Pseudogene | 1 | ICKPLHY / NPFIYS(F/L) | EF427013 |
| Black coucal | CgOR35 | Functional | 1 | ICKPLHY / NPFIYS(F/L) | EF427014 |
| Black coucal | CgOR36 | Functional | 1 | ICKPLHY / NPFIYS(F/L) | EF427015 |
| Black coucal | CgOR37 | Functional | 1 | ICKPLHY / NPFIYS(F/L) | EF427016 |
| Black coucal | CgOR38 | Functional | 1 | ICKPLHY / NPFIYS(F/L) | EF427017 |
| Black coucal | CgOR39 | Pseudogene | 1 | VAICKPLHY / NPFIYS(F/L) | EF427018 |
| Black coucal | CgOR40 | Functional | 1 | VAICKPLHY / NPFIYS(F/L) | EF427019 |
| Black coucal | CgOR41 | Functional | 1 | VAICKPLHY / NPFIYS(F/L) | EF427020 |
| Black coucal | CgOR42 | Functional | 1 | VAICKPLHY / NPFIYS(F/L) | EF427021 |
| Black coucal | CgOR43 | Functional | 1 | AMAYDRY / NPFIYS(F/L) | EF427022 |
| Black coucal | CgOR44 | Functional | 28 | AMAYDRY / PM(L/F)NP | EF427023 |
| Black coucal | CgOR45 | Functional | 7 | MAYDRY(V/L)AIC / PMLNPLIY | EF427024 |
| Black coucal | CgOR46 | Functional | 9 | MAYDRY(V/L)AIC / PMLNPLIY | EF427025 |
| Black coucal | CgOR47 | Functional | 2 | MAYDRY(V/L)AIC / NPFIYS(F/L) | EF427026 |
| Black coucal | CgOR48 | Functional | 1 | MAYDRY(V/L)AIC / NPFIYS(F/L) | EF427027 |
| Black coucal | CgOR49 | Functional | 1 | MAYDRY(V/L)AIC / NPFIYS(F/L) | EF427028 |
| Black coucal | CgOR50 | Functional | 1 | MAYDRY(V/L)AIC / NPFIYS(F/L) | EF427029 |
| Black coucal | CgOR51 | Functional | 1 | MAYDRY(V/L)AIC / NPFIYS(F/L) | EF427030 |
| Black coucal | CgOR52 | Pseudogene | 1 | MAYDRY(V/L)AIC / NPFIYS(F/L) | EF427031 |
| Black coucal | CgOR53 | Pseudogene | 1 | MAYDRY(V/L)AIC / PM(L/F)NP | EF427032 |
| Black coucal | CgOR54 | Functional | 1 | MAYDRY(V/L)AIC / PM(L/F)NP | EF427033 |
| Black coucal | CgOR55 | Functional | 1 | AMAYDRY / NPFIYS(F/L) | EF427034 |
| Black coucal | CgOR56 | Functional | 1 | MAYDRY / PMLNPLIY | EF427035 |
| Black coucal | CgOR57 | Functional | 1 | MAYDRY / NPFIYS(F/L) | EF427036 |
| Black coucal | CgOR58 | Functional | 6 | MAYDRY / NPFIYS(F/L) | EF427037 |
| Black coucal | CgOR59 | Functional | 3 | AMAYDRY / PMLNPLIY | EF427038 |
| Black coucal | CgOR60 | Functional | 1 | AMAYDRY / PMLNPLIY | EF427039 |
| Black coucal | CgOR61 | Functional | 2 | AMAYDRY / NPFIYS(F/L) | EF427040 |
| Snow petrel | PnOR1 | Functional | 2 | ICKPLHY / NPFIYS(F/L) | EF427298 |
| Snow petrel | PnOR2 | Functional | 1 | ICKPLHY / NPFIYS(F/L) | EF427299 |
| Snow petrel | PnOR3 | Pseudogene | 4 | ICKPLHY / NPFIYS(F/L) | EF427300 |
| Snow petrel | PnOR4 | Functional | 1 | ICKPLHY / NPFIYS(F/L) | EF427301 |
| Snow petrel | PnOR5 | Functional | 2 | ICKPLHY / NPFIYS(F/L) | EF427302 |
| Snow petrel | PnOR6 | Functional | 1 | ICKPLHY / NPFIYS(F/L) | EF427303 |
| Snow petrel | PnOR7 | Functional | 2 | ICKPLHY / NPFIYS(F/L) | EF427304 |
| Snow petrel | PnOR8 | Functional | 1 | ICKPLHY / NPFIYS(F/L) | EF427305 |
| Snow petrel | PnOR9 | Functional | 1 | ICKPLHY / NPFIYS(F/L) | EF427306 |
| Snow petrel | PnOR10 | Pseudogene | 2 | ICKPLHY / NPFIYS(F/L) | EF427307 |
| Snow petrel | PnOR11 | Functional | 1 | ICKPLHY / NPFIYS(F/L) | EF427308 |
| Snow petrel | PnOR12 | Functional | 1 | VAICKPLHY / NPFIYS(F/L) | EF427309 |
| Snow petrel | PnOR13 | Functional | 1 | VAICKPLHY / NPFIYS(F/L) | EF427310 |
| Snow petrel | PnOR14 | Functional | 1 | VAICKPLHY / NPFIYS(F/L) | EF427311 |
| Snow petrel | PnOR15 | Functional | 4 | VAICKPLHY / NPFIYS(F/L) | EF427312 |
| Snow petrel | PnOR16 | Functional | 1 | VAICKPLHY / NPFIYS(F/L) | EF427313 |
| Snow petrel | PnOR17 | Functional | 2 | VAICKPLHY / NPFIYS(F/L) | EF427314 |
| Snow petrel | PnOR18 | Functional | 1 | VAICKPLHY / NPFIYS(F/L) | EF427315 |
| Snow petrel | PnOR19 | Functional | 1 | VAICKPLHY / NPFIYS(F/L) | EF427316 |
| Snow petrel | PnOR20 | Functional | 1 | VAICKPLHY / NPFIYS(F/L) | EF427317 |
| Snow petrel | PnOR21 | Pseudogene | 1 | VAICKPLHY / NPFIYS(F/L) | EF427318 |
| Snow petrel | PnOR22 | Pseudogene | 1 | VAICKPLHY / NPFIYS(F/L) | EF427319 |
| Snow petrel | PnOR23 | Functional | 1 | ICKPLHY / NPFIYS(F/L) | EF427320 |
| Snow petrel | PnOR24 | Functional | 2 | VAICKPLHY / NPFIYS(F/L) | EF427321 |
| Snow petrel | PnOR25 | Functional | 1 | VAICKPLHY / NPFIYS(F/L) | EF427322 |
| Snow petrel | PnOR26 | Functional | 1 | VAICKPLHY / NPFIYS(F/L) | EF427323 |
| Snow petrel | PnOR27 | Functional | 11 | AMAYDRY / PMLNPLIY | EF427324 |
| Snow petrel | PnOR28 | Functional | 3 | MAYDRY(V/L)AIC / PM(L/F)NP | EF427325 |
| Snow petrel | PnOR29 | Functional | 1 | MAYDRY / PMLNPLIY | EF427326 |
| Snow petrel | PnOR30 | Functional | 4 | MAYDRY / NPFIYS(F/L) | EF427327 |
| Snow petrel | PnOR31 | Functional | 1 | MAYDRY / PMLNPLIY | EF427328 |
| Snow petrel | PnOR32 | Functional | 3 | AMAYDRY / PM(L/F)NP | EF427329 |
| Snow petrel | PnOR33 | Functional | 1 | MAYDRY / PMLNPLIY | EF427330 |
| Snow petrel | PnOR34 | Functional | 6 | MAYDRY / NPFIYS(F/L) | EF427331 |
| Snow petrel | PnOR36 | Pseudogene | 1 | AMAYDRY / NPFIYS(F/L) | EF427333 |
| Snow petrel | PnOR37 | Functional | 1 | MAYDRY(V/L)AIC / PMLNPLIY | EF427334 |
| Snow petrel | PnOR38 | Functional | 2 | MAYDRY(V/L)AIC / NPFIYS(F/L) | EF427335 |
| Snow petrel | PnOR39 | Functional | 1 | MAYDRY(V/L)AIC / NPFIYS(F/L) | EF427336 |
| Snow petrel | PnOR40 | Functional | 1 | MAYDRY / PMLNPLIY | EF427337 |
| Snow petrel | PnOR41 | Functional | 4 | MAYDRY / NPFIYS(F/L) | EF427338 |
| Snow petrel | PnOR42 | Functional | 1 | AMAYDRY / NPFIYS(F/L) | EF427339 |
| Snow petrel | PnOR43 | Functional | 1 | MAYDRY / NPFIYS(F/L) | EF427340 |
| Snow petrel | PnOR44 | Functional | 1 | AMAYDRY / NPFIYS(F/L) | EF427341 |
| Snow petrel | PnOR45 | Pseudogene | 1 | MAYDRY(V/L)AIC / NPFIYS(F/L) | EF427342 |
| Snow petrel | PnOR46 | Pseudogene | 2 | AMAYDRY / PMLNPLIY | EF427343 |
| Snow petrel | PnOR47 | Functional | 1 | MAYDRY(V/L)AIC / PMLNPLIY | EF427344 |
| Snow petrel | PnOR48 | Functional | 1 | MAYDRY / NPFIYS(F/L) | EF427345 |
| Kakapo | ShOR1 | Pseudogene | 2 | ICKPLHY / NPFIYS(F/L) | EF427113 |
| Kakapo | ShOR2 | Functional | 2 | ICKPLHY / NPFIYS(F/L) | EF427114 |
| Kakapo | ShOR3 | Functional | 1 | ICKPLHY / NPFIYS(F/L) | EF427115 |
| Kakapo | ShOR4 | Functional | 1 | ICKPLHY / NPFIYS(F/L) | EF427116 |
| Kakapo | ShOR5 | Functional | 5 | ICKPLHY / NPFIYS(F/L) | EF427117 |
| Kakapo | ShOR6 | Functional | 1 | ICKPLHY / NPFIYS(F/L) | EF427118 |
| Kakapo | ShOR7 | Functional | 2 | ICKPLHY / NPFIYS(F/L) | EF427119 |
| Kakapo | ShOR8 | Functional | 3 | ICKPLHY / NPFIYS(F/L) | EF427120 |
| Kakapo | ShOR9 | Pseudogene | 1 | ICKPLHY / NPFIYS(F/L) | EF427121 |
| Kakapo | ShOR10 | Functional | 1 | ICKPLHY / NPFIYS(F/L) | EF427122 |
| Kakapo | ShOR11 | Functional | 7 | ICKPLHY / NPFIYS(F/L) | EF427123 |
| Kakapo | ShOR12 | Pseudogene | 1 | VAICKPLHY / NPFIYS(F/L) | EF427124 |
| Kakapo | ShOR13 | Functional | 2 | ICKPLHY / NPFIYS(F/L) | EF427125 |
| Kakapo | ShOR14 | Functional | 1 | ICKPLHY / NPFIYS(F/L) | EF427126 |
| Kakapo | ShOR15 | Functional | 1 | ICKPLHY / NPFIYS(F/L) | EF427127 |
| Kakapo | ShOR16 | Functional | 1 | ICKPLHY / NPFIYS(F/L) | EF427128 |
| Kakapo | ShOR17 | Pseudogene | 4 | ICKPLHY / NPFIYS(F/L) | EF427129 |
| Kakapo | ShOR18 | Functional | 1 | ICKPLHY / NPFIYS(F/L) | EF427130 |
| Kakapo | ShOR19 | Functional | 1 | VAICKPLHY / NPFIYS(F/L) | EF427131 |
| Kakapo | ShOR20 | Pseudogene | 1 | ICKPLHY / NPFIYS(F/L) | EF427132 |
| Kakapo | ShOR21 | Functional | 1 | ICKPLHY / NPFIYS(F/L) | EF427133 |
| Kakapo | ShOR22 | Functional | 1 | ICKPLHY / NPFIYS(F/L) | EF427134 |
| Kakapo | ShOR23 | Functional | 1 | ICKPLHY / NPFIYS(F/L) | EF427135 |
| Kakapo | ShOR24 | Functional | 1 | VAICKPLHY / NPFIYS(F/L) | EF427136 |
| Kakapo | ShOR25 | Functional | 1 | ICKPLHY / NPFIYS(F/L) | EF427137 |
| Kakapo | ShOR26 | Pseudogene | 1 | VAICKPLHY / NPFIYS(F/L) | EF427138 |
| Kakapo | ShOR27 | Functional | 1 | ICKPLHY / NPFIYS(F/L) | EF427139 |
| Kakapo | ShOR28 | Functional | 1 | ICKPLHY / NPFIYS(F/L) | EF427140 |
| Kakapo | ShOR29 | Functional | 1 | ICKPLHY / NPFIYS(F/L) | EF427141 |
| Kakapo | ShOR30 | Pseudogene | 1 | VAICKPLHY / NPFIYS(F/L) | EF427142 |
| Kakapo | ShOR31 | Functional | 1 | VAICKPLHY / NPFIYS(F/L) | EF427143 |
| Kakapo | ShOR32 | Functional | 13 | MAYDRY / PMLNPLIY | EF427144 |
| Kakapo | ShOR33 | Functional | 12 | MAYDRY / NPFIYS(F/L) | EF427145 |
| Kakapo | ShOR34 | Functional | 1 | AMAYDRY / PMLNPLIY | EF427146 |
| Kakapo | ShOR35 | Functional | 1 | AMAYDRY / NPFIYS(F/L) | EF427147 |
| Kakapo | ShOR36 | Functional | 3 | AMAYDRY / PM(L/F)NP | EF427148 |
| Kakapo | ShOR37 | Functional | 3 | MAYDRY(V/L)AIC / PMLNPLIY | EF427149 |
| Kakapo | ShOR38 | Functional | 1 | MAYDRY(V/L)AIC / PMLNPLIY | EF427150 |
| Kakapo | ShOR39 | Functional | 1 | MAYDRY(V/L)AIC / PMLNPLIY | EF427151 |
| Kakapo | ShOR40 | Functional | 1 | MAYDRY(V/L)AIC / NPFIYS(F/L) | EF427152 |
| Kakapo | ShOR41 | Functional | 2 | MAYDRY(V/L)AIC / NPFIYS(F/L) | EF427153 |
| Kakapo | ShOR42 | Pseudogene | 1 | MAYDRY(V/L)AIC / NPFIYS(F/L) | EF427154 |
| Kakapo | ShOR43 | Functional | 1 | MAYDRY(V/L)AIC / NPFIYS(F/L) | EF427155 |
| Kakapo | ShOR44 | Functional | 4 | MAYDRY(V/L)AIC / NPFIYS(F/L) | EF427156 |
| Kakapo | ShOR45 | Functional | 1 | MAYDRY(V/L)AIC / PM(L/F)NP | EF427157 |
| Kakapo | ShOR46 | Functional | 1 | MAYDRY(V/L)AIC / PM(L/F)NP | EF427158 |
| Kakapo | ShOR47 | Pseudogene | 1 | MAYDRY(V/L)AIC / PM(L/F)NP | EF427159 |
| Kakapo | ShOR48 | Functional | 1 | MAYDRY / NPFIYS(F/L) | EF427160 |
| Kakapo | ShOR49 | Pseudogene | 1 | MAYDRY(V/L)AIC / PM(L/F)NP | EF427161 |
| Kakapo | ShOR50 | Functional | 1 | MAYDRY / PMLNPLIY | EF427162 |
| Kakapo | ShOR51 | Functional | 1 | MAYDRY / PMLNPLIY | EF427163 |
| Kakapo | ShOR52 | Functional | 1 | MAYDRY / NPFIYS(F/L) | EF427164 |
| Kakapo | ShOR53 | Functional | 1 | AMAYDRY / NPFIYS(F/L) | EF427165 |
| Kakapo | ShOR54 | Functional | 1 | AMAYDRY / NPFIYS(F/L) | EF427166 |
| Kakapo | ShOR55 | Functional | 1 | AMAYDRY / NPFIYS(F/L) | EF427167 |
| Kakapo | ShOR56 | Functional | 1 | MAYDRY(V/L)AIC / NPFIYS(F/L) | EF427168 |
| Brown kiwi | AaOR1 | Functional | 1 | ICKPLHY / NPFIYS(F/L) | EF427169 |
| Brown kiwi | AaOR2 | Functional | 2 | ICKPLHY / NPFIYS(F/L) | EF427170 |
| Brown kiwi | AaOR3 | Pseudogene | 1 | ICKPLHY / NPFIYS(F/L) | EF427171 |
| Brown kiwi | AaOR4 | Functional | 1 | ICKPLHY / NPFIYS(F/L) | EF427172 |
| Brown kiwi | AaOR5 | Functional | 1 | ICKPLHY / NPFIYS(F/L) | EF427173 |
| Brown kiwi | AaOR6 | Functional | 3 | ICKPLHY / NPFIYS(F/L) | EF427174 |
| Brown kiwi | AaOR7 | Functional | 1 | ICKPLHY / NPFIYS(F/L) | EF427175 |
| Brown kiwi | AaOR8 | Functional | 1 | ICKPLHY / NPFIYS(F/L) | EF427176 |
| Brown kiwi | AaOR9 | Functional | 1 | ICKPLHY / NPFIYS(F/L) | EF427177 |
| Brown kiwi | AaOR10 | Functional | 1 | ICKPLHY / NPFIYS(F/L) | EF427178 |
| Brown kiwi | AaOR11 | Functional | 1 | ICKPLHY / NPFIYS(F/L) | EF427179 |
| Brown kiwi | AaOR12 | Functional | 2 | ICKPLHY / NPFIYS(F/L) | EF427180 |
| Brown kiwi | AaOR13 | Pseudogene | 2 | ICKPLHY / NPFIYS(F/L) | EF427181 |
| Brown kiwi | AaOR14 | Functional | 1 | ICKPLHY / NPFIYS(F/L) | EF427182 |
| Brown kiwi | AaOR15 | Functional | 1 | ICKPLHY / NPFIYS(F/L) | EF427183 |
| Brown kiwi | AaOR16 | Functional | 1 | ICKPLHY / NPFIYS(F/L) | EF427184 |
| Brown kiwi | AaOR17 | Functional | 1 | ICKPLHY / NPFIYS(F/L) | EF427185 |
| Brown kiwi | AaOR18 | Pseudogene | 1 | ICKPLHY / NPFIYS(F/L) | EF427186 |
| Brown kiwi | AaOR19 | Functional | 1 | ICKPLHY / NPFIYS(F/L) | EF427187 |
| Brown kiwi | AaOR20 | Functional | 1 | ICKPLHY / NPFIYS(F/L) | EF427188 |
| Brown kiwi | AaOR21 | Pseudogene | 1 | ICKPLHY / NPFIYS(F/L) | EF427189 |
| Brown kiwi | AaOR22 | Functional | 1 | ICKPLHY / NPFIYS(F/L) | EF427190 |
| Brown kiwi | AaOR23 | Functional | 1 | ICKPLHY / NPFIYS(F/L) | EF427191 |
| Brown kiwi | AaOR24 | Functional | 1 | VAICKPLHY / NPFIYS(F/L) | EF427192 |
| Brown kiwi | AaOR25 | Functional | 1 | ICKPLHY / NPFIYS(F/L) | EF427193 |
| Brown kiwi | AaOR26 | Functional | 1 | VAICKPLHY / NPFIYS(F/L) | EF427194 |
| Brown kiwi | AaOR27 | Pseudogene | 1 | VAICKPLHY / NPFIYS(F/L) | EF427195 |
| Brown kiwi | AaOR28 | Functional | 1 | ICKPLHY / NPFIYS(F/L) | EF427196 |
| Brown kiwi | AaOR29 | Functional | 1 | ICKPLHY / NPFIYS(F/L) | EF427197 |
| Brown kiwi | AaOR30 | Pseudogene | 1 | ICKPLHY / NPFIYS(F/L) | EF427198 |
| Brown kiwi | AaOR31 | Functional | 1 | ICKPLHY / NPFIYS(F/L) | EF427199 |
| Brown kiwi | AaOR32 | Functional | 1 | ICKPLHY / NPFIYS(F/L) | EF427200 |
| Brown kiwi | AaOR33 | Functional | 1 | ICKPLHY / NPFIYS(F/L) | EF427201 |
| Brown kiwi | AaOR34 | Pseudogene | 1 | ICKPLHY / NPFIYS(F/L) | EF427202 |
| Brown kiwi | AaOR35 | Functional | 1 | ICKPLHY / NPFIYS(F/L) | EF427203 |
| Brown kiwi | AaOR36 | Functional | 1 | ICKPLHY / NPFIYS(F/L) | EF427204 |
| Brown kiwi | AaOR37 | Pseudogene | 2 | VAICKPLHY / NPFIYS(F/L) | EF427205 |
| Brown kiwi | AaOR38 | Functional | 1 | VAICKPLHY / NPFIYS(F/L) | EF427206 |
| Brown kiwi | AaOR39 | Functional | 1 | VAICKPLHY / NPFIYS(F/L) | EF427207 |
| Brown kiwi | AaOR40 | Functional | 1 | MAYDRY / NPFIYS(F/L) | EF427208 |
| Brown kiwi | AaOR41 | Functional | 1 | AMAYDRY / PMLNPLIY | EF427209 |
| Brown kiwi | AaOR42 | Pseudogene | 11 | AMAYDRY / PMLNPLIY | EF427210 |
| Brown kiwi | AaOR43 | Functional | 3 | AMAYDRY / NPFIYS(F/L) | EF427211 |
| Brown kiwi | AaOR44 | Functional | 2 | AMAYDRY / NPFIYS(F/L) | EF427212 |
| Brown kiwi | AaOR45 | Functional | 2 | AMAYDRY / NPFIYS(F/L) | EF427213 |
| Brown kiwi | AaOR46 | Functional | 2 | MAYDRY / NPFIYS(F/L) | EF427214 |
| Brown kiwi | AaOR47 | Functional | 3 | AMAYDRY / PM(L/F)NP | EF427215 |
| Brown kiwi | AaOR48 | Functional | 4 | AMAYDRY / PM(L/F)NP | EF427216 |
| Brown kiwi | AaOR49 | Pseudogene | 1 | MAYDRY(V/L)AIC / PMLNPLIY | EF427217 |
| Brown kiwi | AaOR50 | Functional | 2 | MAYDRY(V/L)AIC / PMLNPLIY | EF427218 |
| Brown kiwi | AaOR51 | Pseudogene | 1 | MAYDRY(V/L)AIC / PMLNPLIY | EF427219 |
| Brown kiwi | AaOR52 | Pseudogene | 1 | MAYDRY(V/L)AIC / PMLNPLIY | EF427220 |
| Brown kiwi | AaOR53 | Functional | 1 | MAYDRY(V/L)AIC / NPFIYS(F/L) | EF427221 |
| Brown kiwi | AaOR54 | Functional | 3 | MAYDRY(V/L)AIC / NPFIYS(F/L) | EF427222 |
| Brown kiwi | AaOR55 | Functional | 1 | MAYDRY(V/L)AIC / NPFIYS(F/L) | EF427223 |
| Brown kiwi | AaOR56 | Functional | 1 | MAYDRY(V/L)AIC / NPFIYS(F/L) | EF427224 |
| Brown kiwi | AaOR57 | Functional | 1 | MAYDRY(V/L)AIC / NPFIYS(F/L) | EF427225 |
| Brown kiwi | AaOR58 | Pseudogene | 1 | MAYDRY(V/L)AIC / NPFIYS(F/L) | EF427226 |
| Brown kiwi | AaOR59 | Pseudogene | 2 | MAYDRY(V/L)AIC / NPFIYS(F/L) | EF427227 |
| Brown kiwi | AaOR60 | Functional | 1 | AMAYDRY / NPFIYS(F/L) | EF427228 |
| Brown kiwi | AaOR61 | Pseudogene | 2 | MAYDRY / NPFIYS(F/L) | EF427229 |
| Brown kiwi | AaOR62 | Pseudogene | 1 | MAYDRY / PMLNPLIY | EF427230 |
| Brown kiwi | AaOR63 | Functional | 1 | MAYDRY(V/L)AIC / PMLNPLIY | EF427231 |
| Brown kiwi | AaOR64 | Functional | 1 | MAYDRY(V/L)AIC / PMLNPLIY | EF427232 |
| Brown kiwi | AaOR65 | Functional | 1 | MAYDRY(V/L)AIC / NPFIYS(F/L) | EF427233 |
| Brown kiwi | AaOR66 | Functional | 1 | MAYDRY(V/L)AIC / NPFIYS(F/L) | EF427234 |
| Brown kiwi | AaOR67 | Functional | 1 | MAYDRY(V/L)AIC / PMLNPLIY | EF427235 |
| Brown kiwi | AaOR68 | Functional | 1 | MAYDRY / NPFIYS(F/L) | EF427236 |

**TABLE S2.** Abundance coverage estimators and related statistics for nine avian species. As abundance coverage estimator, ACE_1 was used (Chao & Lee 1992).

| Common name | Estimate | CI (lower)^1^ | CI (upper) ^1^ | SEM^2^ | n^3^ | D^4^ | C^5^ | CV^6^ |
| --- | --- | --- | --- | --- | --- | --- | --- | --- |
| Canary | 166.0 | 106.5 | 225.5 | 59.5 | 101 | 55 | 0.66 | 0.88 |
| Galah | 107.0 | 61.0 | 153.0 | 66.0 | 47 | 26 | 0.66 | 1.09 |
| Blue tit | 218.2 | 134.1 | 302.3 | 84.1 | 99 | 62 | 0.59 | 0.86 |
| Jungle fowl | 637.5 | 416.3 | 858.7 | 405.4 | 125 | 46 | 0.77 | 1.95 |
| Mallard | 430.3 | 178.3 | 682.3 | 252.0 | 113 | 61 | 0.63 | 1.32 |
| *Black coucal* |  |  |  |  | *115* | *61* | *0.565* | *2.561* |
| Snow petrel | 211.6 | 98.8 | 570.3 | 106.2 | 86 | 47 | 0.64 | 1.08 |
| Kakapo | 666.9 | 386.5 | 947.3 | 338.8 | 106 | 56 | 0.60 | 1.53 |
| Brown kiwi | 599.9 | 387.8 | 812.0 | 397.6 | 99 | 68 | 0.48 | 1.20 |

^1^ CI = Confidence interval of the estimate

^2^ SEM = Standard error of the estimate

^3^ n = Number of clones analyzed

^4^ D = Distinct number of cloned genes

^5^ C = Estimation of the sample coverage

^6^ CV = Estimation of the coefficient of variation

### Supplemental references

Chao, A. & Lee, S. M. 1992 Estimating the Number of Classes Via Sample Coverage. *J. Am. Stat. Assoc.* **87**, 210-217.

Sibley, C. G. & Ahlquist, J. E. 1991 *Phylogeny and classification of birds: A Study in Molecular Evolution*. New Haven: Yale University Press.
